# Supplementary material for: Synergistic integration of Netrin and ephrin axon guidance signals by spinal motor neurons
Source: eLife. 2015 Dec 3;4:e10841. doi: 10.7554/eLife.10841 (PMC4764565; doi:10.7554/eLife.10841)
Supplement: Supplementary file 1. — DOI: http://dx.doi.org/10.7554/eLife.10841.019 [file elife-10841-supp1.docx]

| **Supplementary File 1A:**  **Antibodies and Fc reagents used** | |  |  |
| --- | --- | --- | --- |
|  |  |  |  |
| **Antigen / pure protein** | **Source species** | **Dilution** | **Source/reference** |
| EphA4 | Rabbit | 1:1000* | Santa Cruz |
| EphB1 | Goat | 1:500 | Santa Cruz |
| Lmx1b | Guinea pig | 1:4000 | (Kania et al., 2000) |
| neurofilament | Mouse (2H3) | 1:100 | DSHB |
|  | Mouse (3A10) | 1:100 | (Dodd and Jessell, 1988) |
| β-gal | Rabbit | 1:4000 | Rockland |
| Netrin1 | Goat | 1:500 | R&D system |
|  | Mouse | 1:100 | R&D system |
| Neogenin | Goat | 1:100 | R&D system |
| Unc5c | Rabbit | 1:12000 | See Exp. Procedures |
| Isl1/2 | Mouse | 1:100 | DSHB |
|  | Rabbit | 1:2500 | (Tsuchida et al., 1994) |
| DCC | Goat | 1:500 | R&D system |
| HRP | Goat | 1:2000 | Roche |
| Foxp1 | Guinea pig | 1:1000 | (Dasen et al., 2008) |
| GFP | Guinea pig | 1:1000 | AbD Serotec |
| GFP | Rabbit | 1:2000 Western  IP = 2 μg/ip; 1/1000 | Molecular Probes |
| Netrin-1 | Mouse | C_100_ (stripes) = 100 ng/mL  collapse “low” = 300 ng/ml  IP = 250 ng/ml | R&D system |
| ephrin-A5-Fc | Human | C_100_ (stripes) = 10 μg/mL  Collapse “high” = 10 μg/mL  collapse “low” = 1 μg/mL  IP = 1.5 μg/mL | R&D system |
| ephrin-B2-Fc | Mouse | C_100_ (stripes) = 10 μg/mL  Collapse “high” = 10 μg/mL  collapse “low” = 1 μg/mL  IP = 1.5 μg/mL | R&D system |
| ephrin-A3-Fc | Mouse | 1.5 μg/mL | R&D system |
| Fc | Human | 10 μg/mL | R&D system |
| EphB2 | Goat | 1:1000 | R&D system |
| EEA1 | Rabbit | 1:1000 | Abcam |
| anti-Fc | Mouse | 1:4 mass ratio to ephrin | Sigma |
|  | Goat | 1:4 mass ratio to ephrin | Sigma |
| Unc5c | Rabbit | 1:1000 | Abgent |
| Tuj1 | Mouse | 1:1000 | Covance |
| 568-Phalloidin |  | 1:300 | Life Technologies |
| MYC | Mouse | 1:1500 Western | Cell Signaling |
| HA | Mouse | 1: 30 Western | DHSB 12CA5-1 |
| pSFK | Rabbit | 1:1000 | Life Technologies |
| pEphB2 | Rabbit | 1:1000 | (Dalva et al., 2000) |
|  |  |  |  |
| * Concentrations of these antibodies were doubled for in vitro experiments. | | | |

**Supplementary File 1B:**

**Quantifications of Main Figures**

| Fig. 2I | wt: 4.4±2.6% | | *Ntn1^Gt/Gt^*: 4.9±3.6% | |  |
| --- | --- | --- | --- | --- | --- |
|  | *Unc5c^-/-^*: 5.6±0.6% | | *Dcc*^-/-^: 9.7± 2.9% | |  |
| Fig. 2S | wt: 3.9±1.8% | | *Ntn1^Gt/Gt^*: 12.6±4.4% | |  |
|  | *Unc5c^-/-^*: 40.4±18.0% | | *Dcc^-/-^*: 3.3±1.8% | |  |
| Fig. 3A | 1^st^ Fc: 48.1±5.1% | | 2^nd^ Fc: 51.9±5.1% | |  |
| Fig. 3B | N: 74.8±5.6% | | Fc: 25.2±5.6% | |  |
| Fig. 3C | N: 53.1±4.4% | | Fc: 46.9±4.4% | |  |
| Fig. 3D | N: 47.6±1.1% | | Fc: 52.4±1.1% | |  |
| Fig. 3E | N: 66.2±5.8% | | Fc: 33.8±5.8% | |  |
| Fig. 3F | 1^st^ Fc: 51.3±3.8% | | 2^nd^ Fc: 48.7±3.8% | |  |
| Fig. 3G | N: 24.2±4.0% | | Fc: 76.2±4.0% | |  |
| Fig. 3H | N: 48.4±5.0% | | Fc: 51.6±5.0% | |  |
| Fig. 3I | N: 28.4±7.4% | | Fc: 71.6±7.4% | |  |
| Fig. 3J | N: 30.3±7.1% | | Fc: 69.7±7.1% | |  |
| Fig. 3K | N: 58.9±9.2% | | Fc: 41.1±9.2% | |  |
| Fig. 3L | N: 27.1±3.5% | | Fc: 72.9±3.5% | |  |
| Fig. 4A | d: 52.3±6.1% | | v: 47.7±6.1% | |  |
| Fig. 4B | d: 71.7±6.1% | | v: 28.3±6.1% | |  |
| Fig. 4C | d: 40.7±8.3% | | v: 59.3±8.3% | |  |
| Fig. 4D | d: 36.3±8.9% | | v: 63.7±8.9% | |  |
| Fig. 4E | d: 19.8±6.0% | | v: 80.2±6.0% | |  |
| Fig. 4F | d: 51.8±4.5% | | v: 48.2±4.5% | |  |
| Fig. 4G | d: 46.5±5.8% | | v: 53.5±5.8% | |  |
| Fig. 4H | d: 47.8±6.3% | | v: 52.2±6.3% | |  |
| Fig. 4I | d: 25.6±8.1% | | v: 74.4±8.1% | |  |
| Fig. 5A | eB2: 21.8±7.7% | | Fc: 78.2±7.7% | |  |
| Fig. 5B | eB2+N: 9.5±8.5% | | Fc: 90.5±8.5% | |  |
| Fig. 5C | N: 49.1±10.1% | | Fc: 50.9±10.1% | |  |
| Fig. 5D | eB2: 50.2±9.2% | | Fc: 49.8±9.2% | |  |
| Fig. 5E | eB2+N: 24.2±12.5% | | Fc: 75.8±12.5% | |  |
| Fig. 5F | eA­­5: 23.2±10.1% | | Fc: 76.8±10.1% | |  |
| Fig. 5G | eA5: 13.1±12.7% | | N: 86.9±12.7% | |  |
| Fig. 5H | N: 50.2±8.5% | | Fc: 49.8±8.5% | |  |
| Fig. 5I | eA5: 51.2±7.5% | | Fc: 48.8±7.5% | |  |
| Fig. 5J | eA5: 25.1±10.7% | | N: 74.9±10.7% | |  |
| Fig. 6B | Fc: 17.87±9.01% | | High eB2: 61.76±12.83% | |  |
|  | Low eB2: 31.62±12.32% | | Low Netrin-1: 22.89±4.24% | |  |
|  | eB2+N: 72.93±17.55% | |  | |  |
| Fig. 6J | Fc: 0.32±0.10 | | eB2: 0.32±0.01 | |  |
|  | Netrin-1: 0.27±0.02 | | eB2+N: 0.28±0.08 | |  |
| Fig. 6N | | pEphB2 signal increase | |  | |
|  | | eB2: 1 | | eB2 + Netrin-1: 1.23±0.26 | |
| Fig. 7G | | Fc: 1±1.3 | | Low eB2: 1.4±1.4 | |
|  | | High eB2: 1.5±2 | | Low Netrin-1: 0.5±0.2 | |
|  | | High Netrin-1: 1±0.9 | | Low eB2+Netrin-1: 4.6±2.6 | |
| Fig. 7I | | Fc (DMSO): 39.3±7.8% | | Fc (SU6656): 36±4% | |
|  | | eB2+N (DMSO): 74.5±5.4% | | eB2+N (SU6656): 68.7±4.5% | |
|  | | 1/10 eB2+N (DMSO): 60.8±7.6% | | 1/10 eB2+N (SU6656): 49.5±14% | |
|  | | 1/30 eB2+N (DMSO): 60.3±7.6% | | 1/30 eB2+N (SU6656): 42.5±8.8% | |
| Fig. 7J | | eB2: 27.3±7.5% | | Fc: 72.7±7.5% | |
| Fig. 7K | | eB2: 52.5±8.7% | | Fc: 47.5±8.7% | |
| Fig. 7L | | N: 27.9±9.1% | | Fc: 72.1±9.1% | |
| Fig. 7M | | N: 43.7±11.3% | | Fc: 56.3±11.3% | |
| Fig. 7N | | eB2+N: 16.7±5.6% | | Fc: 83.3±5.6% | |
| Fig. 7O | | eB2+N: 35.5±10.7% | | Fc: 64.5±10.7% | |
| Fig. 7P | | eB2+N: 28.9±7.9% | | Fc: 71.1±7.9% | |
| Fig. 7Q | | eB2+N: 46.4±9.3% | | Fc: 53.6±9.3% | |
|  | |  | |  | |

**Supplementary File 1C:**

**Quantifications of Figure supplements**

| Fig. 2S1I | wt: d 2.2±1.2% | wt: v 97.8±1.2% |
| --- | --- | --- |
|  | *Ntn1^Gt/Gt^*: d 14.2±9.8% | *Ntn1^Gt/Gt^*: v 85.8±9.8% |
|  | *Unc5c^-/-^*: d 64.5±7.4% | *Unc5c^-/-^*: v 35.5±7.4% |
|  | *Dcc^-/-^*: d 1.2±1.5% | *Dcc^-/-^*: v 98.8±1.5% |
| Fig. 2S2B | wt LMCm: 50.1±4.8 | wt LMCl: 43.3±4.4 |
|  | *Ntn1^Gt/Gt^* LMCm: 49.1±3.7 | *Ntn1^Gt/Gt^* LMCl: 46.6±3.5 |
|  | *Unc5c^-/-^* LMCm: 46.1±3.6 | *Unc5c^-/-^* LMCl: 43.9±3.8 |
|  | *Dcc^-/-^* LMCm: 46.8±5.1 | *Dcc^-/-^* LMCl: 50.1±5.7 |
| Fig. 2S2E | wt V: 4.4±2.6% | wt D: 3.9±1.8% |
|  | *Neo^Gt/Gt^* V: 10.6±6.2% | *Neo^Gt/Gt^* D: 6.6±4.3% |
|  | *Dcc^-/-^ Neo^Gt/Gt^* V: 7.1±2.8% | *Dcc^-/-^ Neo^Gt/Gt^* D: 6.2±1.0% |
|  | *Unc5a^-/-^* V: 5.4±6.2% | *Unc5a^-/-^* D: 5.8±3.9% |
|  | *Unc5a^-/-^ Unc5c^-/-^* V: 5.9±4.9% | *Unc5a^-/-^ Unc5c^-/-^* D: 44.8±13.7% |
|  | *Dscam^-/-^* V: 7.5±5.2% | *Dscam^-/-^* D: 6.6±2.3% |
| Fig. 2S2F | wt V: 2.7±0.6% | wt D: 0.7±0.5% |
|  | *Ntn1^Gt/Gt^* V: 7.7±6.4% | *Ntn1^Gt/Gt^* D: 2.7±2.8% |
|  | *Unc5c*^-/-^ V: 4.7±1.7% | *Unc5c*^-/-^ D: 24.9±22.1% |
|  | *Dcc^-/-^* V: 4.4±2.2% | *Dcc^-/-^* V: 3.6±3.4% |
|  | *Neo^Gt/Gt^* V: 3.3±3.6% | *Neo^Gt/Gt^* D: 4.6±2.1% |
|  | *Dcc^-/-^ Neo^Gt/Gt^* V: 6.6±2.8% | *Dcc^-/-^ Neo^Gt/Gt^* D: 1.6±1.7% |
|  | *Unc5a^-/-^* V: 2.1±1.9% | *Unc5a^-/-^* D: 3.9±3.2% |
|  | *Unc5a^-/-^ Unc5c^-/-^* V: 18.2±5.4% | *Unc5a^-/-^ Unc5c^-/-^* D: 30.1±2.2% |
|  | *Dscam^-/-^* V: 6.3±4.0% | *Dscam^-/-^* D: 4.9±3.3% |
| Fig. 3S1D | eA5: 25.8±3.4% | Fc: 74.2±3.4% |
| Fig. 3S1E | eA5: 25.1±6.2% | Fc: 74.9±6.2% |
| Fig. 3S1F | *Isl1*^+^*Unc5c*^+^: 95.6±3.8% |  |
| Fig. 3S1G | *Isl1*^+^*Unc5c*^+^: 95.3±2.9% |  |
| Fig. 3S1H | *Isl1*^+^*Ephb1*^+^: 94.1±3.5% |  |
| Fig. 4S1B | *GFP*: 1.06±0.15 | *[Unc5c]siRNA*: 0.51±0.06 |
| Fig. 4S1C | *GFP*: 71.3±5.3 | *[Unc5c]siRNA*: 69.1±9.4 |
| Fig. 4S1D | *GFP* m: 47.9±4.2% | *GFP* l: 52.1±4.2% |
|  | *[Unc5c]siRNA* m: 50.9±8.5% | *[Unc5c]siRNA* l: 49.1±8.5% |
| Fig. 4S1E | *GFP* m: 51.1±11.9% | *GFP* l: 48.9±11.9% |
|  | *[Unc5c]siRNA* m: 48.1±10.5% | *[Unc5c]siRNA* l: 51.9±10.5% |
| Fig. 4S1H | *GFP*: 75.0±10.2 | *Unc5c*+*GFP*: 74.0±7.5 |
| Fig. 4S1I | *GFP* m: 50.7±2.8% | *GFP* l: 49.3±2.8% |
|  | *Unc5c*+*GFP* m: 51.4±3.8% | *Unc5c*+*GFP* l: 48.5±3.8% |
| Fig. 4S1J | *GFP* m: 52.5±8.2% | *GFP* l: 47.5±8.2% |
|  | *Unc5c*+*GFP* m: 50.9±8.5% | *Unc5c*+*GFP* l: 49.1±8.5% |
| Fig. 4S1M | *GFP*: 0.2847  *Ephb2*: 0.3345 | *Unc5c*: 0.3077  *Foxp1*: 0.9255 |
| Fig. 4S1O | Low *GFP*: 7.2±0.4% (16 misprojecting out of 218) | Low *Ephb2-GFP*: 4.3±4.7% (12 misprojecting out of 265) |
|  | Low *Unc5c* + *GFP*: 7.5±4.6% (29 misprojecting out of 345) | Low *EphB2-GFP* + *Unc5c*: 16.7±2.7% (92 misprojecting out of 538) |
| Fig. 5S1A | N: 58.6±11.5% | Fc: 41.4±11.5% |
| Fig. 5S1B | eA5: 45.3±7.7% | Fc: 54.7±7.7% |
| Fig. 5S1C | eA5: 14.1±11.2% | N: 85.9±11.2% |
| Fig. 5S1D | N: 52.3±9.3% | Fc: 47.7±9.3% |
| Fig. 5S1E | eA5: 46.1±8.1% | Fc: 53.9±8.1% |
| Fig. 5S1F | eA5: 25.3±11.2% | N: 74.7±11.2% |
| Fig. 5S1G | N: 48.6±7.4% | Fc: 51.4±7.4% |
| Fig. 5S1H | eB2: 43.1±9.1% | Fc: 56.9±9.1% |
| Fig. 5S1I | eB2+N: 11.0±9.8% | Fc: 89.0±9.8% |
| Fig. 5S1J | N: 48.2±8.1% | Fc: 51.8±8.1% |
| Fig. 5S1K | eB2: 44.7±6.9% | Fc: 55.3±6.9% |
| Fig. 5S1L | eB2+N: 21.0±12.2% | Fc: 79.0±12.2% |
| Fig. 6S1B | Fc: 105.9±26.45 μm^2^ | eB2: 125.8±17.75 μm^2^ |
|  | Netrin-1: 123.8±35.64 μm^2^ | eB2+N: 120.3±28.8 μm^2^ |
| Fig. 6S1C | Fc: 37.50±3.44% | eB2: 19.06±15.40% |
|  | Netrin-1: 35.61±7.00% | eB2+N: 29.31±19.56% |
| Fig. 6S1D | Fc: 23.59±15.63% | eB2: 9.90±1.255% |
|  | Netrin-1: 20.96±6.28% | eB2+N: 14.44±10.83% |
| Fig. 6S1F | Fc: 1±0 | eB2: 1±0 |
|  | Netrin-1: 0.99±0.0005 | eB2+N: 0.99±0.0127 |
| Fig. 6S1G | Fc: 95.46±18.62 μm^2^ | eB2: 102.2±12.10 μm^2^ |
|  | Netrin-1: 94.86±4.53 μm^2^ | eB2+N: 103.4±3.764 μm^2^ |
|  |  | Perm: 125.6±25.27 μm^2^ |
| Fig. 6S1H | Fc: 10.65±4.34% | eB2: 11.89±10.83% |
|  | Netrin-1: 11.56±11.51% | eB2+N: 10.44±5.01% |
|  |  | Perm: 45.03±9.63% |
| Fig. 6S1I | Fc: 24.56±18.54% | eB2: 26.80±9.26% |
|  | Netrin-1: 19.35±4.95% | eB2+N: 32.57±5.48% |
|  |  | Perm: 76.05±14.43% |
| Fig. 6S1J | Fc: 0.98±0.42% | eB2: 0.85±0.59% |
|  | Netrin-1: 0.65±0.34% | eB2+N: 1.08±0.61% |
|  |  | Perm: 10.53±4.57% |
| Fig. 6S1N | Unc5c co-ip (fold change/Fc) | Fc: 1 |
|  | Netrin-1: 1.05±0.44 | eB2: 23.37±2.41 |
|  | eB2+N: 22.2±4.23 |  |
| Fig. 6S1O | Unc5c co-ip  (fold change/EphB2-GFP) |  |
|  | EphB2-GFP: 1±0 | EphB2-KD-GFP: 0.47±0.14 |
| Fig. 7S1J | Fc (labeled 0): 0.12±0.07% | 1 ng eB2: 0.16±0.18% |
|  | 10 ng eB2: 0.04±0.05% | 100 ng eB2: 0.46±0.41% |
|  | 1,000 ng eB2: 0.78±0.63% | 10,000 ng eB2: 1.96±0.94% |
| Fig. 7S1K | Fc: 1±0.6 | Low Netrin-1: 0.87±0.7 |
|  | Low eB2: 6.6±5.3 | Low eB2+Netrin-1: 8.4±7.03 |
|  | High eB2: 16.5±7.9 |  |
| Fig. 7S1L | Fc: 33.6±11.7 μm^2^ | Low eB2: 40±13.6 μm^2^ |
|  | High eB2: 47.2±13.6 μm^2^ | Low Netrin-1: 29.8±6.1 μm^2^ |
|  | High Netrin-1: 40.3±2.3 μm^2^ | Low eB2+Netrin-1: 38.4±10.9 μm^2^ |
| Fig. 7S1M | Fc + DMSO: 29.3±6.03% | Fc + SU6656: 27.7±3.79% |
|  | eB2 + DMSO: 61.7±2.52% | eB2 + SU6656: 39.3±8.08% |
|  |  |  |
|  |  |  |
